# Pituitary metastases from neuroendocrine neoplasms: case report and narrative review

**DOI:** 10.1007/s11102-021-01178-9

**Published:** 2021-08-03

**Authors:** Alberto Ragni, Alice Nervo, Mauro Papotti, Nunzia Prencipe, Francesca Retta, Daniela Rosso, Marta Cacciani, Giuseppe Zamboni, Francesco Zenga, Silvia Uccella, Paola Cassoni, Marco Gallo, Alessandro Piovesan, Emanuela Arvat

**Affiliations:** 1 Oncological Endocrinology Unit, Department of Medical Sciences, University of Turin, Turin, Italy; 2 Pathology Division, Department of Oncology, University of Turin, Turin, Italy; 3 Endocrinology and Metabolism Unit, Department of Medical Sciences, University of Turin, Turin, Italy; 4 Endocrinology Unit, Department of Biomedical, Metabolic and Neural Sciences, University of Modena and Reggio Emilia, Modena, Italy; 5 Pathology Division, Ospedale Sacro Cuore Don Calabria, Negrar and University of Verona, Verona, Italy; 6 Neurosurgery Unit, Department of Neurosciences and Mental Health, University of Turin, Turin, Italy; 7 Pathology Unit, Department of Medicine and Surgery, University of Insubria, Varese, Italy; 8 Pathology Division, Department of Medical Sciences, University of Turin, Turin, Italy; 9 Endocrine and Metabolic Diseases Unit, SS. Antonio E Biagio E Cesare Arrigo Hospital, Alessandria, Italy

**Keywords:** Neuroendocrine tumours, NET, Neuroendocrine neoplasm, Hypophysis, Sellar metastases, Hypopituitarism

## Abstract

**Purpose:**

Pituitary metastases (PM) are uncommon findings and are mainly derived from breast and lung cancers. No extensive review of PM from neuroendocrine neoplasms (NENs) is on record. Here we describe a clinical case of PM from pancreatic NEN and review the clinical features of PM from NENs reported in the literature.

**Methods:**

A case of PM from a pancreatic NEN followed at our institution is described. We also reviewed the 43 cases of PM from NENs reported in the literature.

**Results:**

A 59-year old female patient, previously submitted to duodeno-cephalo-pancreasectomy for a well-differentiated pancreatic NEN, with known hepatic metastases, underwent a ^68^ Ga-DOTATOC PET/CT that revealed an uptake in the pituitary gland. A subsequent MRI displayed a pituitary lesion, with suprasellar extension. After a hormonal and genetic diagnostic workup that excluded the diagnosis of MEN 1, the worsening of headache and visual impairment and the growth of the lesion lead to its surgical removal. A pituitary localization of the pancreatic NEN was identified. Regarding the published cases of PM from NENs, the most common tumour type was small cell lung cancer (SCLC), accounting for nearly half of the cases, followed by bronchial and pancreatic well differentiated NENs. The most frequent symptom was a variable degree of visual impairment, while headache was reported in half of the cases. Partial or total anterior hypopituitarism was present in approximately three quarters of the cases, while diabetes insipidus was less common. The most frequent treatment for PM was surgical resection, followed by radiotherapy and chemotherapy. The clinical outcome was in line with previous reports of PM from solid tumours, with a median survival of 14 months. Surgery of PM was associated with prolonged survival.

**Conclusions:**

PM from NENs have clinical features similar to metastases derived from other solid tumours, albeit the involvement of the anterior pituitary seems more frequent; a thorough pituitary hormonal evaluation is mandatory, after focused radiological studies, particularly if a surgical approach is considered. The optimal management of PM remains disputed and seems mainly driven by the aggressiveness of the primary tumour and the presence of symptoms. In well-differentiated NENs, particularly in the case of symptomatic PM, surgical removal may be a reasonable approach.

## Introduction

Pituitary metastases (PM) are uncommon findings and their reported incidence is variable. Being often asymptomatic, their prevalence is higher in autopsy series [1]; moreover, they can be incidentally found during imaging workup in patients with advanced neoplastic disease [2]. Given the absence of pathognomonic radiological or clinical findings and the risk of hormonal disarrangement derived from pituitary tissue invasion, differentiating PM from pituitary adenomas can be difficult [2, 3]. Breast and lung cancer are the tumours that most frequently metastasize to the pituitary gland [1]. However, PM derived from neuroendocrine neoplasms (NENs) have been increasingly reported especially in the last decades [4–6]. While extensive reviews regarding clinical presentations and management of PM from several solid tumours have already been published [1, 3], no studies specifically regarding PM from NENs are on record. Here we reported the case of a PM from a pancreatic NEN managed at our centre and we reviewed the published literature regarding PM from NENs (both single case reports and case series), describing their main clinical, diagnostic and therapeutic features.

## Case presentation

**Fig. 1:**
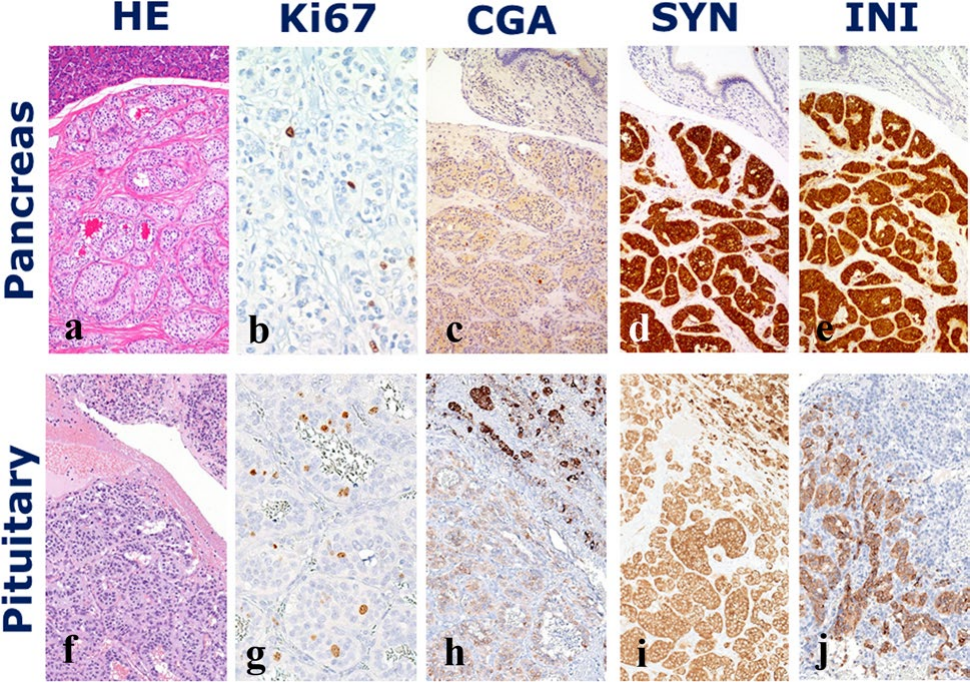
Pancreatic NEN progressed with metastasis to the pituitary gland. **a** microscopic features of the pancreatic NEN with an organoid growth made of occasionally clear cells with scant atypias; **b** the ki-67 proliferative index was low (1%) and the tumour was graded G1; **c**, **d** chromogranin A and synaptophysin were diffusely positive; **e** the tumour was also strongly reactive for inhibin. The resected PM had a similar architecture (**f**), but tumour cells had no clear cytoplasm, and rather showed an increased ki-67 proliferative activity (8%) **g** tumour cells also shared the immunoprofile of the primary pancreatic tumour, with diffuse reactivity for chromogranin A (**h**), synaptophysin (**i**) and inhibin (**l**). *NEN* neuroendocrine neoplasm, *PM* pituitary metastasis

We report the case of a 59-years old female patient who was followed-up at our tertiary centre for metastatic pancreatic NEN. The tumour was incidentally detected in 2008 by an abdominal CT scan and, therefore, the patient underwent duodenocephalopancreasectomy with lymphadenectomy. The pathology report was consistent with a G1 well-differentiated pancreatic neuroendocrine tumour (NET) (Ki-67 index: 4%) with metastasis in 1 out of 32 excised lymph nodes. Tumour cells did not express pancreatic hormones but intense and diffuse immunoreactivity for the general neuroendocrine marker chromogranin A reactivity was observed. In addition, inhibin expression was detected, as rarely occurring in NETs with clear cell changes [7] (see Fig. 1). Given the absence of residual disease, no adjuvant therapy with somatostatin analogues was administered at that time.

**Fig. 2:**
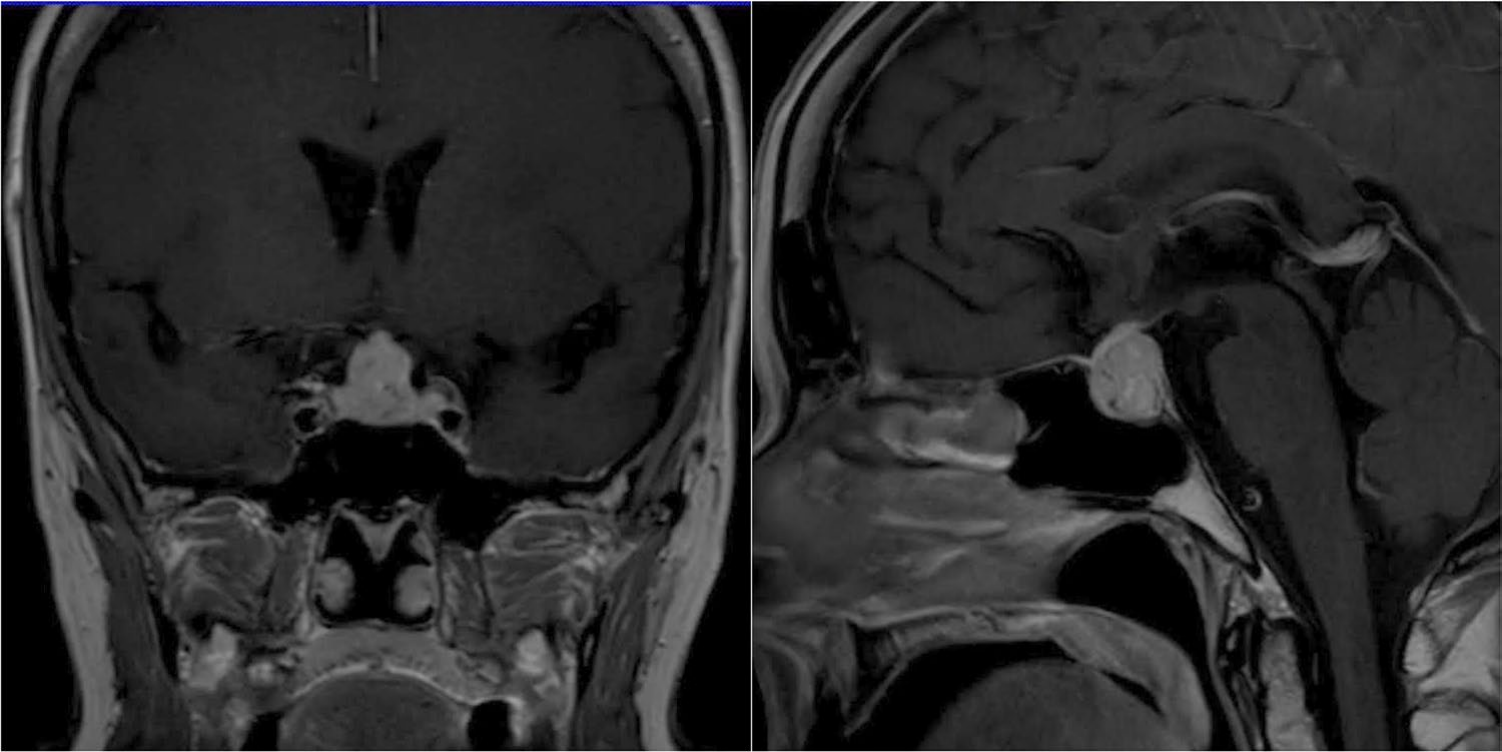
Coronal (left) and sagittal (right) pre-operative MRI scans showing the inhomogeneous sellar lesion with suprasellar extension. *MRI* magnetic resonance imaging

In 2017, after 9 years of complete remission, the tumour relapsed and a small lesion in the liver with several enlarged abdominal lymph nodes was detected by means of MRI. At that time, the patient refused therapy with somatostatin analogues. In June 2018, a restaging ^68^ Ga-DOTATOC PET/CT confirmed the presence of the known metastatic lesions, apparently stable in size after 11 months, and revealed a slightly increased accumulation of the radiotracer in the pituitary gland. At the time, the patient complained about the onset of bilateral galactorrhea but denied any headache, polyuria or polydipsia. The subsequent MRI scan described the presence of a large (20-mm in its largest diameter) pituitary lesion, that was supposed to be a pituitary adenoma. In particular, the lesion had a suprasellar extension with compression of the optic chiasm and the suprasellar cistern, and displacement of the pituitary stalk. It was dyshomogeneously hypointense and hyperintense in T1-and T2-weighted sequences, respectively, and showed a strong and premature contrast enhancement (see Fig. 2). The visual field campimetry showed bitemporal impairment. The biochemical and hormonal tests (see Table 1) demonstrated normal levels of sodium, potassium, calcium, parathyroid hormone (PTH), gonadotropins (considering the postmenopausal condition) and IGF-1 by age; in contrast, prolactin was moderately increased and the evaluation of the pituitary-thyroid axis was consistent with secondary hypothyroidism. The normal response of cortisol to 1 µg Cosyntropin ruled out a possible secondary adrenal insufficiency. We started treatment with cabergoline and levothyroxine and galactorrhea subsequently disappeared. A genetic test for multiple endocrine neoplasia (MEN) 1 syndrome was performed, but mutations in the *MEN1* gene were ruled out.

**Table 1:** Hormonal and electrolyte values before and after TSS

Hormones	Reference range	Pre-surgery	Post-surgery (1 month post-TSS)	Last follow-up (12 months post-TSS)
TSH, µUI/ml	0.27–4.20	2.64	< 0.005	< 0.005
fT4, pg/ml	9.3–17.0	8.6	8.3^a^	11.3^a^
fT3, pg/ml	2.6–4.4	–	1.5^a^	2.1^a^
ACTH, pg/ml	< 46	17	< 5	< 5
Basal cortisol, µg/l	62–194	122	8.1	1.9^b^
Stimulated* cortisol, µg/l	> 180	221	–	–
Prolactin, ng/ml	4.8–23.3	119	< 0.1	–
IGF-1, ng/ml	65–320	83	–	–
Sodium, mmol/l	135–145	144	149	140
Potassium, mmol/l	3.5–5.0	4.6	5.3	4.6
pOsm, mOsm/Kg	278–305	301	300^c^	293^c^
uOsm, mOsm/Kg	50–1400	112	232^c^	218^c^
PTH, pg/ml	15–57	48	–	–
Calcium, mmol/l	2.2–2.6	2.25	–	–

*TSS* trans-sphenoidal surgery

*1 µg cosynthropin test

^a^during replacement therapy with levothyroxine

^b^after 24 h from the last dose of corticosteroid replacement therapy

^c^during replacement therapy with desmopressin

Given the gradual onset of headache, the worsening of the visual impairment, and the radiological evidence of progressive enlargement of the pituitary mass at a subsequent MRI scan after 6 months, a 3D extended endoscopic endonasal approach to sellar and suprasellar region was performed [8] in June 2019. Postoperative clinical course was characterized by the development of diabetes insipidus (DI) that required therapy with desmopressin. The histopathological examination of the surgical specimen was consistent with a pituitary localization of a well-differentiated NEN, but the differential diagnosis of a pituitary adenoma from a metastatic NET was not possible on morphological ground, only (see Fig. 1). Immunohistochemical stainings for pituitary hormones (prolactin, ACTH, GH, FSH, LH, TSH) as well as those for pituitary markers TPIT, Pit1 and GATA3 were all negative. Despite the pituitary tumour did not contain clear cells, inhibin immunostaining was diffusely positive, with a pattern similar to that of the primary pancreatic NET. The proliferative index (assessed by the MIB1 monoclonal antibody to Ki-67) was 8%, almost double than that of the primary pancreatic NET. Pathology of the resected tumour was eventually interpreted as a PM of the pancreatic G2 NET resected 11 years earlier.

The post-surgical hormonal tests (see Table 1) confirmed the secondary hypothyroidism and revealed the onset of secondary hypocortisolism. Hence, glucocorticoid hormonal replacement therapy was added to the levothyroxine and desmopressin treatment.

Given the progression of the liver disease, therapy with monthly octreotide was started after surgery. Three and six months after surgery, follow-up MRI scans showed no sign of residual tissue in the sellar region. Unfortunately, no improvement was demonstrated at the visual field campimetry evaluation. One year after surgery, the patient underwent restaging scans with both ^68^ Ga- and ^18^F-FDG PET/CT, which showed no uptake in the pituitary region and stability of the known liver and lymph node metastases.

The patient is currently in good clinical conditions and pituitary deficiencies are well controlled by replacement therapy two years after pituitary surgery and 13 years after pancreatic NET resection. Her therapy continues with monthly octreotide with good tolerance.

## Literature review

**Fig. 3:**
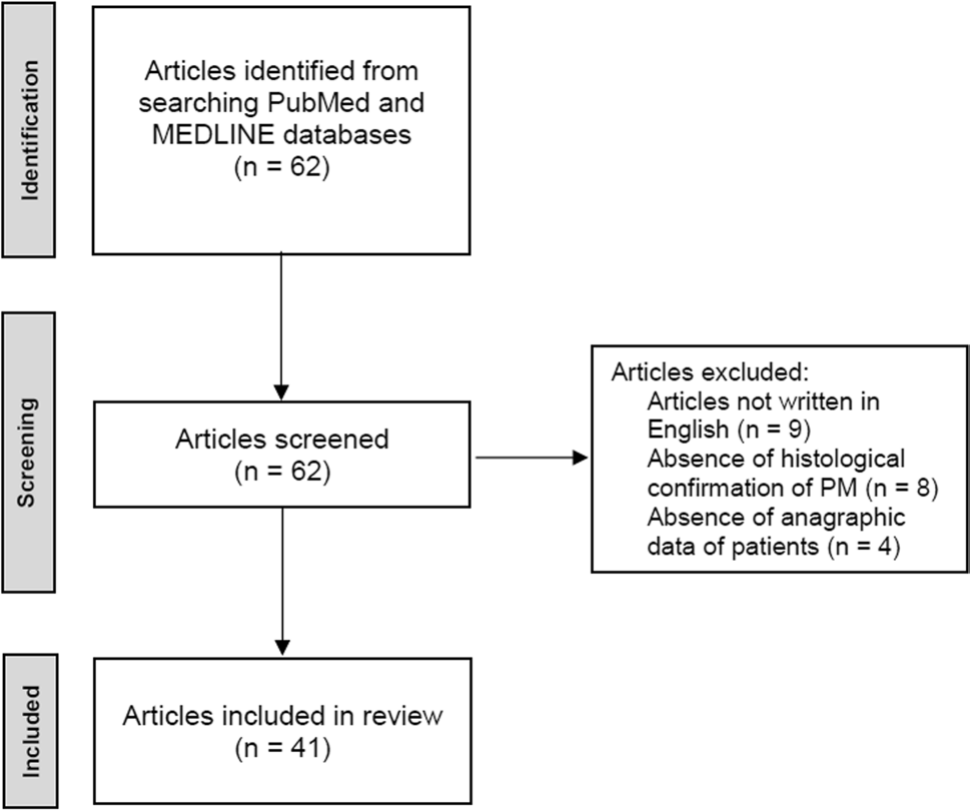
Flowchart for the literature review

We thoroughly reviewed all cases of PM from NENs available from the literature. We searched PubMed and MEDLINE database using the following, variously combined, keywords: “pituitary metastasis”, “sellar metastasis”, “neuroendocrine tumour”, “neuroendocrine neoplasm”, “neuroendocrine carcinoma”, “small cell lung cancer”, “carcinoid tumour”. A review of the abstract of each retrieved study was then carried out. Only publications with abstract and/or full text in English language were included. Studies in which the histological diagnosis of the primary NEN and of PM was lacking were excluded. Figure 3 shows the process of identification, screening and exclusion of the retrieved articles.

For each case, the following data were gathered: year of publication, gender and age of the patient, primary tumour and PM histology, known metastatic disease before discovery of PM, presenting signs and symptoms, and treatment of PM. In those PM who were not surgically treated, histology of PM was obtained through either autopsy or biopsy of the lesion. When available, also hormonal alterations, radiological features at the time of diagnosis as well as survival data were collected and analysed. Survival analysis was performed using the Kaplan–Meier method and survival distribution comparison was done using the logrank test.

Finally, 42 reported cases of pituitary masses histopathologically consistent with a pituitary localization of NENs were found between 1984 and 2020 [4–6, 9–46]; by adding our patient, a total of 43 cases were analysed. The relevant data are summarized in Table 2. The average age of patients at PM diagnosis was 58 years (range 23–82 years) with a slight female predominance (56%).

**Table 2:** Demographic, pathological, clinical, hormonal, imaging and therapeutic characteristics of the 43 reported cases of PM from NENs

Total number of cases	n = 43 (%)
Sex	n = 43 (%)
M	19 (44)
F	24 (56)
Median age (y)	58
Primary NEN histology	n = 43 (%)
SCLC	21 (49)
Atypical lung carcinoid	6 (14)
Pancreatic NEN	4 (9)
MTC	3 (7)
Thymic carcinoid	2 (5)
Unknown primary NEN	2 (5)
Breast NEN	2 (5)
Ileal NEN	1 (2)
Merkel cell carcinoma	1 (2)
Lung LCNEC	1 (2)
PM as first manifestations of NEN	23 (53)
Clinical presentation	n = 41 (%)
Visual field deficit	27 (66)
Headache	20 (49)
Constitutional symptoms (asthenia, weight loss)	18 (44)
Other ocular symptoms (diplopia, blurred vision)	18 (44)
Ocular palsy	9 (22)
Other neurological symptoms (gait disturbance, confusion, memory loss)	6 (15)
Hormonal alterations	n = 37 (%)
Hypopituitarism (deficit of at least one pituitary axis)	27 (73)
Hyperprolactinemia	21 (57)
Gonadotropins deficit	19 (51)
ACTH deficit	15 (40)
TSH deficit	15 (40)
DI	13 (35)
Panhypopituitarism (complete anterior pituitary deficit + DI)	6 (16)
Imaging characteristics	n = 36 (%)
Suprasellar extension	30 (83)
Compression/displacement of pituitary stalk	20 (56)
Bone erosion	9 (25)
Invasion of cavernous sinus	7 (19)
Involvement of other brain structures	7 (19)
Thickening/involvement of pituitary stalk	4 (11)
Therapy	n = 43 (%)
Surgery	32 (74)
Radiotherapy	22 (51)
Chemotherapy	19 (44)
PRRT	2 (5)
Median survival [months, (95% CI)]	14 (9–21)

*PM* pituitary metastases, *NEN* neuroendocrine neoplasm, *SCLC* small cell lung cancer, *MTC* medullary thyroid cancer, *LCNEC* large cell neuroendocrine carcinoma, *DI* diabetes insipidus, *PRRT* peptide receptor radionuclide therapy, *IQR* interquartile range

Small cell lung cancer (SCLC) was the most represented primary NEN (49%), followed by atypical bronchial carcinoid (14%), pancreatic well-differentiated NENs (9%), and medullary thyroid cancer (MTC; 7%). The primary site of the remaining cases is listed in Table 2. Only one patient (with metastatic MTC) had a definitive diagnosis of multiple endocrine neoplasia (MEN) 2B syndrome [32]. No patients with MEN 1 syndrome were present among the cases retrieved.

PM appeared as a manifestation of an already known metastatic disease in only 23% of patients. In the remaining cases, mainly consisting of SCLC, PM was the first presentation of a neoplastic disease of unknown primary.

Hormonal data were available for 86% (37/43) of patients. Hypopituitarism (defined as a deficit of at least one of the adenohypophysis hormonal axes) was the most common endocrine alteration, being present in 73% of patients. Hyperprolactinemia was found in 57% of cases. The gonadal axis was involved in nearly half of patients (51%), while the corticotroph and the thyrotroph axes were less frequently impaired (both 40%). DI was reported in approximately one third of the patients (35%). Panhypopituitarism (i.e. the presence of complete anterior pituitary deficit and DI) was present in 16% of cases only.

A paraneoplastic syndrome related to the ectopic secretion of pituitary peptides from NEN cells was detected in six cases (Cushing syndrome in four and acromegaly in two patients) [13, 16, 21, 28, 29, 46]. Interestingly, in five other cases, PM developed within a pituitary adenoma (two non-functioning adenomas, one prolactinoma, one GH-secreting and one ACTH-secreting adenoma) [12, 30, 32, 34, 43].

Data on neurological or ocular symptoms at PM diagnosis were available for 95% (41/43) patients. Visual alterations were the most frequent clinical manifestation, being present in all but six patients. Specifically, visual field impairment was present in 66% of patients, 44% reported diplopia or blurred vision, and 22% had paralysis of at least one oculomotor nerve. Headache was reported in 49% of patients.

Relevant radiological features detected by MRI and/or CT scan were reported in 84% (36/43) patients. Most of them (83%) had a suprasellar extension; a displacement and/or a compression of the optic chiasm was radiologically found in more than half cases (56%). Bone erosion was observed in one case, while only 19% showed an invasion of the cavernous sinus. Four radiological reports [11, 19, 21, 28] described a thickening or an involvement of the pituitary stalk. In a minority of patients, the authors reported the involvement of other encephalic structures, such as the compression of the third ventricle (n = 4) [22, 25, 36, 39], of the optic nerve (n = 2) [13, 25] or the extension into the prepontine and premesencephalic cisterns (n = 1) [22].

When available, the signal intensity of PM on the T1-weighted and T2-weighted sequences of the MRI scan was variable among the reports. In two cases, the loss of the expected T1-hyperintense signal of the posterior lobe was observed [11, 19]. After paramagnetic contrast injection, MRI scan demonstrated a homogeneous enhancement for some lesions, while an inhomogeneous enhancement was described by other studies. The pituitary mass was associated to other central nervous system (CNS) metastatic lesions in 17% of cases. Moreover, a rapid PM growth was reported in three patients who performed two MRI scans at a close time interval [6, 36, 41]. Two PM appeared calcified [6, 40] and one caused a fibrotic reaction around the sella turcica [23].

Nearly three quarters (74%) of patients underwent surgical resection of PM, mainly through a trans-sphenoidal approach; a craniotomic approach was performed only in three patients (in all cases during the 1980s) and one patient had a trans-ciliary approach. Among patients not surgically treated, but having a histological diagnosis based on the biopsy, all but one underwent radiotherapy (RT) or chemotherapy (CT) treatment regimens. Overall, RT and CT were employed in 51% and 44% of patients, respectively. Only in two patients peptide receptor radionuclide therapy (PRRT) was used in combination with other treatments.

Regarding clinical, hormonal and/or local improvement after therapy of PM, data were lacking for the majority of patients. Among the cases with available data on neurological outcome (30%, 13/43), 77% showed clinical improvement of neurological symptoms; among them, almost all the patients were surgically treated with only one case that did not undergo surgery, but was treated with RT and CT. Regarding ocular symptoms (data available for 53% of patients, 23/43), some improvement after therapy was seen in 61% of patients. All but two of these patients had undergone surgical resection of PM and the majority was also treated with RT. Given the lack of data, it was not possible to carry out an analysis regarding hormonal improvement after PM therapy.

**Fig. 4:**
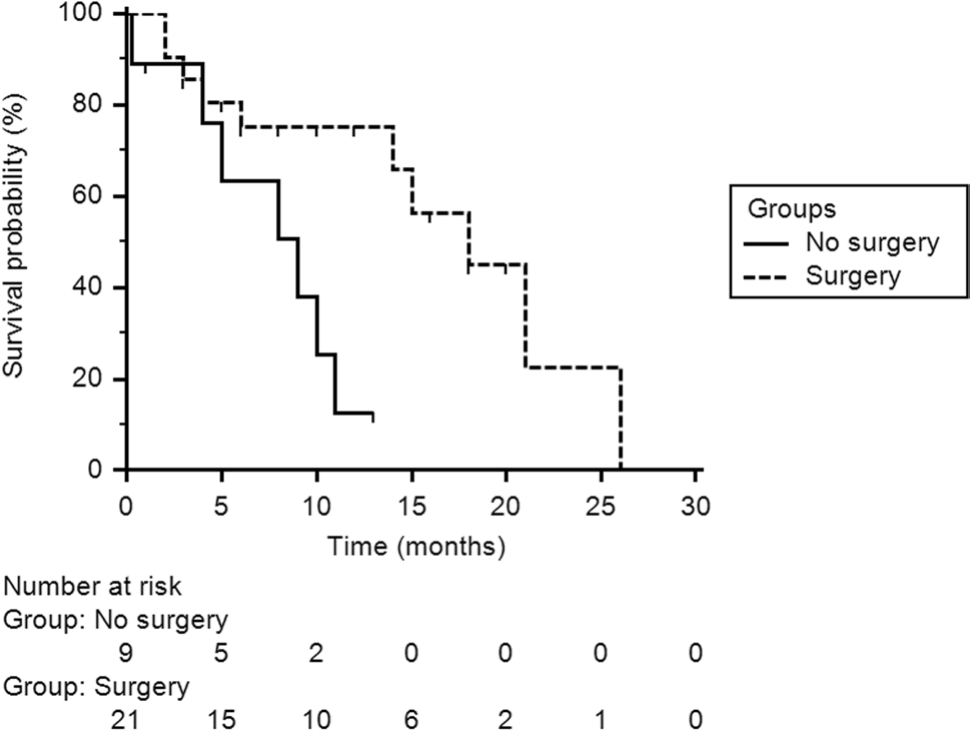
Kaplan–Meier survival analysis comparing outcomes between surgically-treated (dashed line) versus non surgically-treated (solid line) patients with PM. *PM* pituitary metastases

Survival data were available for 70% of patients (30/43). The median survival from PM diagnosis was 14 months (95% CI 9–21 months); five patients survived more than 18 months. Surgery was associated with prolonged survival (see Fig. 4): patients who underwent surgical resection of PM had a median survival of 18 months (95% CI 14.0–26.0), compared to 9 months (95% CI 4.0–11.0) for patients that were not surgically treated (p = 0.09). No difference in survival was noted in patients with SCLC compared with patients with NENs from other histology.

## Discussion

We presented a rare case of PM from a pancreatic NET, which gives us the opportunity to speculate on the tools for distinguishing this lesion from a pituitary adenoma and to revise the literature on metastatic localizations of NENs in the pituitary gland.

PM are rare findings, even in patients with an advanced neoplastic disease. In general, approximately 5% of patients with a known history of malignancy are thought to have a latent PM [47], with the highest percentages in patients with breast cancer [1, 48, 49]. The abundant blood supply of the pituitary gland may facilitate hematogenic metastatic spreading and seeding [50]. As observed in five of the cases included in this review, PM can even develop inside a pituitary adenoma (so called “collision tumours”) that could be hormonally active [32].

Compared to the published reviews on PM from NENs [4–6], the present review on 43 cases (42 from the literature and one presented in this review) by covering a time span of 36 years, allows to better evaluate the clinical features of patients suffering from NEN-derived PM.

Among the cases of PM from NENs, the most common primary tumour was SCLC in line with recently published studies [5, 6]. This finding is somewhat expected, given the aggressive clinical behaviour of this type of tumour and its high metastatic potential, especially to the CNS [51]. In previous studies regarding PM from any primary tumour location, they represented the first manifestation of neoplastic disease in approximately 20–30% of cases [1, 38, 52, 53]. Conversely, in the population analysed in this review, PM was the first manifestation of malignancy in the majority of patients. Most of these cases were represented by SCLC, notoriously characterized by a rapid metastatic spreading.

Regarding symptoms, prevalence of DI and hyperprolactinemia in patients with PM from NEN appears to be in line with other reports regarding PM from other solid tumours [38, 52, 53]. Conversely, in patients with PM from NEN, hypopituitarism, in comparison with recent large reviews in PM from solid tumours [1, 38, 53], was a more common alteration; in this review, the most involved pituitary axis was the gonadal one, followed by secondary hypoadrenalism and hypothyroidism. These findings are consistent with previous observations, although only a small number of patients were tested for hypogonadism in those series [52].

The diagnosis and clinical management of PM, especially from NENs, can be challenging also due to the possible coexistence of paraneoplastic syndromes, as seen in this review. Moreover, when evaluating a sellar mass in a patient with a known NEN, the possibility of a diagnosis of MEN syndrome should be taken into consideration. The currently reported case had only moderately increased PRL levels associated with secondary hypothyroidism. A treatment with cabergoline and levothyroxine was started and mutations in the *MEN1* gene were ruled out.

Also the prevalence of visual alterations and cranial nerve palsy were similar in our analysis compared with previous reports [37, 38, 52, 54]. Given their rarity in pituitary adenomas, some Authors proposed that in the presence of a pituitary mass, either DI or cranial nerve palsies should favour a diagnosis of PM rather than pituitary adenoma, especially if rapidly progressive and affecting patients older than 50 years [1].

MRI is the gold standard for the study of sellar lesions. Although several findings might point toward a diagnosis of PM, radiological techniques are usually unable to clearly distinguish between PM and pituitary adenomas [5]. Moreover, the published reports did not always include a detailed neuroimaging description, limiting the definition of a specific radiological pattern for NEN-derived PM as opposed to pituitary adenomas.

In previous reports, PM have been described as dumbbell-shaped intrasellar and suprasellar lesions, with a clear indentation at the level of the diaphragma sellae on sagittal images. However, this presentation might also be seen in case of benign tumours [37, 54].

According to other studies about PM, MRI generally revealed an isointense or hypointense sellar mass on T1-weighted and hyperintense on T2-weighted images, with homogeneous enhancement after gadolinium. However, neither of these findings has been demonstrated to be highly specific for PM [54]. In this series, the signal intensity of the pituitary lesion on the MRI did not help in the differential diagnosis of the pituitary mass. Some lesions were characterized by inhomogeneous enhancement, probably as a result of intralesional haemorrhage or necrosis. Moreover, the loss of the posterior lobe brightness has been described in two of the reported cases, maybe due to tumour infiltration, similarly to what has been anecdotally reported in literature [37].

The suprasellar extension of the pituitary mass was reported in the majority of the cases included in our review, in accordance with the available data for PM from other tumours [3]. Many of the selected cases also described an involvement of the optic chiasm, with subsequent relevant clinical implications, as observed in our patient.

Being a malignant lesion, tumour growth rate of PM is expected to be higher than for pituitary adenomas [3], also in well-differentiated NENs. Indeed, a rapid growth was reported also among the cases in the current review (including our patient), when serial MRIs were performed at short temporal distance. Interestingly, in our case, the Ki67 proliferation index was higher in the PM than in the primary pancreatic NET. This finding may reflect a tendency of the tumour to progress towards a higher grade, along time, and may have a prognostic meaning [55].

Overall, the differential diagnosis of a sellar mass should include the possibility of PM, especially in case of an enhancing, rapidly-growing suprasellar mass with signs of neurological or hormonal impairment (hypopituitarism, diabetes insipidus, cranial nerve palsy) in a patient aged more than 50 years [56, 57].

There are no specific recommendations for the treatment of sellar masses suspicious for PM. Given the frequent suprasellar extension and local invasiveness, a complete surgical excision is frequently not achievable [58]. Nonetheless, surgical treatment of PM (mainly through a trans-sphenoidal route) could have a role in symptoms palliation [59, 60]. No significant improvement in survival outcomes has been reported after surgery in some cohorts [58, 61], probably because surgical indications had been considered only for rapidly growing and symptomatic PM from more aggressive tumours.

In this review, we found that surgical resection of PM was associated with an improvement in survival. By analysing the limited data of the low number of cases retrieved, it appears that this survival advantage was not related to differences in patients and disease characteristics between the two groups. Nonetheless, it could be postulated that localized or oligometastatic disease and general good clinical condition may support a more aggressive choice of treatment, therefore favouring the surgical approach and a better survival. Although the small number of cases and the retrospective nature of the analysis are clear limits of this analysis, this finding could make surgery a reasonable approach not only for symptoms palliation but also for prolonging survival.

Regarding histopathology, high grade neuroendocrine carcinomas of small and large cells can easily be classified, but the correct distinction of a pituitary adenoma from a metastatic well-differentiated NEN is challenging, since the morphological features of these neoplasms are largely overlapping in the various locations. In such context, a carefully selected immunohistochemical panel is crucial for a good differential diagnosis. Specifically, an algorithmic approach is indicated including, as a first step, pituitary-specific transcription factors (Pit1, TPIT, SF1 and GATA3), as well as TTF1 (for lung NENs and MTC) and CDX2 (for intestinal NENs). Subsequently, the refinement of the diagnosis may benefit of immunostainings for site- and cell type-specific hormonal products and other markers [62]. In the current case, pituitary-related transcription factors and pituitary hormones were not expressed and this was in favour of a metastatic localization of the known pancreatic NET. The rare expression of inhibin, generally associated to NETs with clear cell changes [7] was observed also in the pituitary tumour, further supporting the metastatic nature of the lesion.

Among the therapeutic strategies for PM, RT is another valuable option, usually employed when surgery is not indicated or in the post-operative setting. A recent systematic review showed significant survival advantage in patients treated with RT, in particular with the stereotactic technique [61].

In our series, only two patients with PM from NENs have been treated with PRRT [5, 23], with a substantial radiological and clinical response in one of them [5]. PRRT is a novel treatment option shown to produce substantial responses in patients with metastatic well-differentiated NENs associated to a significant disease burden [63]*.* While no study specifically focused on the response of PM to PRRT was designed, some reports regarding the use of PRRT in the setting of aggressive pituitary tumours are available [64].

Despite the various treatment options available, PM from solid tumours remains a condition with a poor prognosis: previous studies showed a median survival for PM of 13.6 months [38]. Data from this review, with a median survival of 14 months, are in line with previous reports on PM from all solid tumours.

In conclusion, PM is an uncommon manifestation of NENs, and it should be suspected in the presence of hormonal disarrangement and/or ocular or neurological symptoms in patients with previous diagnosis of NEN. Their features are mainly similar to those of PM deriving from other solid tumours. However, PM from NENs seem to show a more frequent involvement of the anterior pituitary rather than the neurohypophysis. Therefore, a thorough hormonal evaluation, including all the pituitary axes, is essential; in particular, secondary adrenal insufficiency must be ruled out given its life-threatening risk. The differential diagnosis from pituitary adenoma is not easy, considering the overlapping morphology with, at least, well-differentiated NEN; thus, a proper use of specific histopathologic markers is needed. Clearly, the high growth rate and the presence of concomitant brain lesions should raise suspicion of PM. Although reported survival outcomes remain poor, surgical resection may be considered in selected patients.

## Data Availability

The data used to support the findings of this study are available from the corresponding author upon request.

## References

[1] He W, Chen F, Dalm B (2015). Metastatic involvement of the pituitary gland: a systematic review with pooled individual patient data analysis. Pituitary.

[2] Goulart CR, Upadhyay S, Ditzel Filho LFS (2017). Newly diagnosed sellar tumors in patients with cancer: a diagnostic challenge and management dilemma. World Neurosurg.

[3] Al-Aridi R, El Sibai K, Fu P (2014). Clinical and biochemical characteristic features of metastatic cancer to the sella turcica: an analytical review. Pituitary.

[4] de Siqueira PF, Mathez ALG, Pedretti DB, Abucham J (2015). Pituitary metastasis of lung neuroendocrine carcinoma: case report and literature review. Arch Endocrinol Metab.

[5] Goglia U, Ferone D, Sidoti M (2008). Treatment of a pituitary metastasis from a neuroendocrine tumour: case report and literature review. Pituitary.

[6] Moshkin O, Rotondo F, Scheithauer BW (2012). Bronchial carcinoid tumors metastatic to the sella turcica and review of the literature. Pituitary.

[7] Gucer H, Szentgyorgyi E, Ezzat S (2013). Inhibin-expressing clear cell neuroendocrine tumor of the ampulla: an unusual presentation of von Hippel-Lindau disease. Virchows Arch.

[8] Pennacchietti V, Garzaro M, Grottoli S (2016). Three-dimensional endoscopic endonasal approach and outcomes in sellar lesions: a single-center experience of 104 cases. World Neurosurg.

[9] Suganuma H, Yoshimi T, Kita T (1994). Rare case with metastatic involvement of hypothalamo-pituitary and pineal body presenting as hypopituitarism and diabetes insipidus. Intern Med.

[10] Aaberg TM, Kay M, Sternau L (1995). Metastatic tumors to the pituitary. Am J Ophthalmol.

[11] Koshimoto Y, Maeda M, Naiki H (1995). MR of pituitary metastasis in a patient with diabetes insipidus. Am J Neuroradiol.

[12] Abe T, Matsumoto K, Iida M (1997). Malignant carcinoid tumor of the anterior mediastinum metastasis to a prolactin-secreting pituitary adenoma: a case report. Surg Neurol.

[13] Genka S, Soeda H, Takahashi M (1995). Acromegaly, diabetes insipidus, and visual loss caused by metastatic growth hormone-releasing hormone-producing malignant pancreatic endocrine tumor in the pituitary gland: Case report. J Neurosurg.

[14] Hopster DJ, Robinson SF, Chadwick L, Geddes JF (1997). Widespread neuroendocrine malignancy within the central nervous system: a diagnostic conundrum. J Clin Pathol.

[15] Huang MC, Lee LS, Ho DMT (2001). A metastatic pituitary carcinoid tumor successfully treated with gamma knife radiosurgery. Chinese Med J.

[16] Isowa N, Nakamura T, Yamazaki F (2003). Thymic atypical carcinoid with cushing’s syndrome manifesting in the terminal stage. Japanese J Thorac Cardiovasc Surg.

[17] Shimon I, Hadani M, Nass D, Zwas ST (2004). Malignant bronchial carcinoid tumor metastatic to the pituitary in a thyroid carcinoma patient: Successful treatment with surgery, radiotherapy and somatostatin analog. Pituitary.

[18] Weilbaecher C, Patwardhan RV, Fowler M (2004). Metastatic lesions involving the sella: report of three cases and review of the literature. Neurol India.

[19] Harzallah L, Migaw H, Harzallah F, Kraiem C (2005). Diabetes insipidus and panhypopituitarism revealing pituitary metastasis of small cell lung carcinoma: A case report. Ann Endocrinol (Paris).

[20] Jonnakuty CG, Mezitis SGE (2007). Pulmonary atypical carcinoid tumor with metastatic involvement of the pituitary gland causing functional hypopituitarism. Endocr Pract.

[21] Nasr C, Mason A, Mayberg M (2006). Acromegaly and somatotroph hyperplasia with adenomatous transformation due to pituitary metastasis of a growth hormone-releasing hormone-secreting pulmonary endocrine carcinoma. J Clin Endocrinol Metab.

[22] Takei H, Buckleair L, Goodman JC, Powell SZ (2007). Intraoperative cytologic diagnosis of symptomatic carcinoma (Pulmonary small cell carcinoma) metastatic to the pituitary gland: a case report. Acta Cytol.

[23] Gur C, Lalazar G, Salmon A (2008). Metastatic pancreatic neuroendocrine tumor presenting as a pituitary space occupying lesion: a case report. Pituitary.

[24] Bhatoe HS, Badwal S, Dutta V, Kannan N (2008). Pituitary metastasis from medullary carcinoma of thyroid: case report and review of literature. J Neurooncol.

[25] Santarpia L, Gagel RF, Sherman SI (2009). Diabetes insipidus and panhypopituitarism due to intrasellar metastasis from medullary thyroid cancer. Head Neck.

[26] Feletti A, Marton E, Rossi S (2010). Pituitary metastasis of merkel cell carcinoma. J Neurooncol.

[27] Luu ST, Billing K, Crompton JL (2010). Clinicopathological correlation in pituitary gland metastasis presenting as anterior visual pathway compression. J Clin Neurosci.

[28] Fridley J, Adams G, Rao V (2011). Small cell lung cancer metastasis in the pituitary gland presenting with seizures and headache. J Clin Neurosci.

[29] Chandra V, Mcdonald LW, Anderson RJ (1984). Metastatic small cell carcinoma of the lung presenting as pituitary apoplexy and Cushing ’ ssyndrome. J Neuro-oncol.

[30] Nassiri F, Cusimano M, Rotondo F (2012). Neuroendocrine tumor of unknown origin metastasizing to a growth hormone-secreting pituitary adenoma. World Neurosurg.

[31] Conway A, Wiernik A, Rawal A (2012). Occult primary medullary thyroid carcinoma presenting with pituitary and parotid metastases: Case report and review of the literature. Endocr Pathol.

[32] Campbell D, Amponsah N, Mott R, Ellis T (2012). Carcinoid tumor of the lung metastatic to a previously identified pituitary adenoma. J Surg Case Reports.

[33] Mathioudakis N, Quinones-Hinojosa A, Salvatori R, Basaria S (2012). A lifelong smoker with hypopituitarism: rethinking the hypothesis of a tumor in the hypophysis. Case Rep Med.

[34] Senetta R, Castellano I, Garbossa D (2013). Pituitary metastasis of an unknown neuroendocrine breast carcinoma mimicking a pituitary adenoma. Pathology.

[35] Sogani J, Yang W, Lavi E (2014). Sellar collision tumor involving metastatic lung cancer and pituitary adenoma: radiologic-pathologic correlation and review of the literature. Clin Imaging.

[36] Little EW, Kazmi KS, Queenan JV, Katsetos CD (2016). Synchronous bifocal metastatic involvement of pituitary and pineal glands unmasking small cell lung cancer. Clin Neuropathol.

[37] Castle-Kirszbaum M, Goldschlager T, Ho B (2018). Twelve cases of pituitary metastasis: a case series and review of the literature. Pituitary.

[38] Javanbakht A, D’Apuzzo M, Badie B, Salehian B (2018). Pituitary metastasis: A rare condition. Endocr Connect.

[39] Kałużny M, Zieliński G, Maksymowicz M, Bolanowski M (2019). Hypopituitarism secondary to pituitary metastasis from small cell lung cancer. Polish Arch Intern Med.

[40] Rossi ML, Bevan JS, Fleming KA, Cruz-Sanchez F (1988). Pituitary metastasis from malignant bronchial carcinoid. Tumori.

[41] Hu H, Sengupta A, Bowes D (2020). Pituitary metastasis of pulmonary large cell neuroendocrine carcinoma: a case report. Cureus.

[42] Kimura A, Kuroiwa A, Teramoto A (1988). Metastatic pituitary tumor presenting as bitemporal visual field defects. Neurol Med Chir (Tokyo).

[43] Ramsay JA, Kovacs K, Scheithauer BW (1988). Metastatic carcinoma to pituitary adenomas: a report of two cases. Exp Clin Endocrinol Diabetes.

[44] Allen EM, Kannan SR, Powell A (1989). Infundibular metastasis and panhypopituitarism. J Natl Med Assoc.

[45] Paulus P, Paridaens R, Mockel J (1990). Argyrophilic breast carcinoma, single metastasis to the pituitary gland. Bull Cancer.

[46] Tabata M, Ohnoshi T, Ueoka H (1993). A case of small cell lung cancer associated with diabetes insipidus and Cushing’s syndrome. Nihon Kyobu Shikkan Gakkai Zasshi.

[47] Moreno-Perez O, Peiró FM, López P (2007). An isolated pituitary metastasis as presentation of a differentiated hepatocellular carcinoma mimicking a nonfunctioning macroadenoma. J Endocrinol Invest.

[48] de la Monte SM, Hutchins GM, Moore GW (1984). Endocrine organ metastases from breast carcinoma. Am J Pathol.

[49] Fortunati N, Felicetti F, Donadio M (2015). Pituitary lesions in breast cancer patients: a report of three cases. Oncol Lett.

[50] Angelousi A, Alexandraki KI, Kyriakopoulos G (2020). Neoplastic metastases to the endocrine glands. Endocr Relat Cancer.

[51] van Meerbeeck JP, Fennell DA, De Ruysscher DKM (2011). Small-cell lung cancer. Lancet (London, England).

[52] Schill F, Nilsson M, Olsson DS (2019). Pituitary metastases: a nationwide study on current characteristics with special reference to breast cancer. J Clin Endocrinol Metab.

[53] Heshmati HM, Scheithauer BW, Young WF (2002). Metastases to the pituitary gland. Endocrinologist.

[54] Komninos J, Vlassopoulou V, Protopapa D (2004). Tumors metastatic to the pituitary gland: case report and literature review. J Clin Endocrinol Metab.

[55] Richards-Taylor S, Tilley C, Jaynes E (2017). Clinically significant differences in Ki-67 proliferation index between primary and metastases in resected pancreatic neuroendocrine tumors. Pancreas.

[56] Freda PU, Post KD (1999). Differential diagnosis of sellar masses. Endocrinol Metab Clin North Am.

[57] Famini P, Maya MM, Melmed S (2011). Pituitary magnetic resonance imaging for sellar and parasellar masses: ten-year experience in 2598 patients. J Clin Endocrinol Metab.

[58] Gilard V, Alexandru C, Proust F (2016). Pituitary metastasis: Is there still a place for neurosurgical treatment?. J Neurooncol.

[59] Feiz-Erfan I, Rao G, White WL, McCutcheon IE (2008). Efficacy of trans-septal trans-sphenoidal surgey in correcting visual symptoms caused by Hematogenous metastases to the sella and pituitary gland. Skull Base.

[60] Patel KR, Zheng J, Tabar V (2020). Extended survival after surgical resection for pituitary metastases: clinical features, management, and outcomes of metastatic disease to the sella. Oncologist.

[61] Ng S, Fomekong F, Delabar V (2020). Current status and treatment modalities in metastases to the pituitary: a systematic review. J Neurooncol.

[62] Uccella S, Asa SL, Mete O (2021). Metastatic neuroendocrine neoplasms of unknown primary site. The Spectrum of Neuroendocrine Neoplasia.

[63] Saravana-Bawan B, Bajwa A, Paterson J (2019). Efficacy of 177Lu peptide receptor radionuclide therapy for the treatment of neuroendocrine tumors. Clin Nucl Med.

[64] Giuffrida G, Ferraù F, Laudicella R (2019). Peptide receptor radionuclide therapy for aggressive pituitary tumors: a monocentric experience. Endocr Connect.

